# Differentiation of Geographic Origin of South African Wines from Austrian Wines by IRMS and SNIF-NMR

**DOI:** 10.3390/foods12061175

**Published:** 2023-03-10

**Authors:** Micha Horacek, Helene Nieuwoudt, Florian F. Bauer, Bahareh Bagheri, Mathabatha E. Setati

**Affiliations:** 1 Department of Lithospheric Research, Vienna University, 1090 Vienna, Austria; 2 South African Grape and Wine Research Institute, Department of Viticulture and Enology, Stellenbosch University, Stellenbosch 7602, South Africa

**Keywords:** isotope ratio mass spectrometry, Specific Natural Isotope Fractionation Nuclear Magnetic Resonance, precipitation, irrigation, drought stress, climate, environmental conditions

## Abstract

Geographic origin and terroir are very important parameters for wine and significantly impact price. Incorrect declarations are known to occur intentionally to increase profit, thus, measures for control are required. Accompanying paperwork has been shown to be unreliable, thus, control of the product itself is required. Here we investigate and compare the stable isotope pattern of South African (Western Cape Province) wine, and evaluate its potential for discrimination from Central European/Austrian wine. The results show that the isotope values of the investigated South African wine samples differ significantly from the values of average Austrian (Central European) wines. Thus, a differentiation of the products from these two regions by stable isotope analysis is generally straightforward. However, the data suggest that vintages from years with exceptionally hot and dry summer weather in Europe may reduce the differentiation between these regions. Therefore, this method is a potent tool for the discrimination of Austrian (Central European) and South African wines under current climatic conditions, although drier and hotter summer weather in Europe, which is likely to occur more frequently due to global climate change, may require further method adjustments in the future.

## 1. Introduction

Wine is a globally relevant lifestyle and luxury food commodity, and consequently, a very important product in agro-economy. In addition, it is one of the food commodities most tightly connected to terroir and geographic origin, as both are among the main parameters relevant for the marketing and esteem—and thus pricing—of wine. 

Wine producers, similarly to producers of other food commodities, can pursue two different kind of main marketing strategies, either the development of an individual brand (name of the winery or a wine), or the marketing of wine regions (protected designation of origin (PDO)). The latter strategy is supported by the EU and many PDO wine regions have been already applied. For further insight, please refer to the literature [1,2,3]. Since terroir and origin significantly influence pricing (e.g., see sparkling wine from Champagne, France in comparison to elsewhere), the wine industry faces an ever-present risk of fraud concerning declarations of origin. Thus, there is the need to control the declared geographic origin. Conventionally, control is based on shipping documents. However, these often have been found to be too unreliable. Therefore, methods to detect/identify the origin and control of declared geographic origins based on analysis of the product (wine) itself are necessary. 

Two main techniques can determine wine origin: measurement of the stable isotope ratios of hydrogen, carbon and oxygen using isotope ratio mass spectrometry (IRMS), and Specific Natural Isotope Fractionation Nuclear Magnetic Resonance (SNIF-NMR) [4,5,6,7,8,9]. Both methods have been initially applied to control wine authenticity due to repeated cases of adulteration of wine, which, besides being a health risk, threatened the consumer confidence in wine. Therefore, the EU started in 1991 the EU-wine database to enable and enforce the control of wine authenticity [10]. Since then each wine-producing EU-country produces each year a certain number of authentic wine samples, analyzes them for their IRMS and SNIF-NMR values and feeds these results into the database. Eventually, it was demonstrated that the wine data used for the control of authenticity could also be used for the control of the declared geographic origin [6,7,8,9,11,12,13,14,15,16,17,18,19,20]. The isotope and NMR-data is influenced by the ambient environmental conditions existing at the respective vineyards (also called terroir). The main influencing factors are temperature and precipitation [12]. Thus, the obtained data gives indications concerning these environmental conditions. The carbon isotope value (δ^13^C) is mainly influenced by water availability, the oxygen isotope value (δ^18^O) mainly by the temperature and the isotope ratio of the water that the plants take up (plant-water), (D/H)I indicates the botanical origin of the fermented sugar, and (D/H)II is dominantly influenced by the isotope composition of the plant-water, and thus similarly to δ^18^O [8].

Control of declared geographic origin is important to protect the local wine production of a country from cheaper wine incorrectly labelled as of local origin. However, non-EU wine samples are not currently analyzed, thus, wine studies such as the present one are required to evaluate the possibilities for the differentiation of non-EU from EU (e.g., Austrian) wines. Similarly, and for the same reasons, the South African government aims to be able to differentiate local wines from foreign ones to protect local producers and consumers, considering that the South African wine sector is an important part of the South African economy.

More recently, the control of geographic origin by strontium isotopes has been repeatedly proposed. This method can be powerful, and has been demonstrated to potentially be very successful [21,22,23,24]. An added advantage is that annual collection and analysis of reference samples would no longer be necessary; however, limitations have been documented [25,26,27,28]. The data suggest that heterogeneous bedrock geology in areas where the geographic origin should be controlled might limit the relevance of the Sr-system concerning the control of geographic origin to specific cases and questions. Other proposed methods for the control and differentiation of geographic origin are based on the additional measurement of element concentrations in wine [23,29,30,31]. However, there exists no general agreement about the main factors influencing the elemental patterns, as Leder et al., 2021 [30], relate these patterns mainly to enological practices, while Griboff et al., 2021 [31], (and other researchers, e.g., [32]) identify the geographic origin (bedrock) as a dominant factor [28]. 

In the present study, we analyze the light stable isotopes of C and O by IRMS, and by SNIF-NMR (D/H)I and (D/H)II, which are the site-specific (methyl- and methylene-position) hydrogen isotope ratios of ethanol (e.g., Christoph et al., 2015 [8]). We assume that differentiation of wine from South Africa and Austria (Figure S1) should be possible (for some indicative preliminary results see Christoph et al., 2015 [8]), without the need for annual reference samples for the exact definition of the vintage for differentiation (as the control of wine origin usually relies on the comparison of commercial samples with reference wine data of the same vintage), due to the difference in climatic conditions and the large geographical distance. Still, Horacek et al., 2021 [20], showed that wines of very different environments (Austria–Argentina) have overlapping isotope signatures, even though preliminary data by Christoph et al., 2015 [8], indicated no overlap.

In this study, we analyzed ca. 50 South African wine samples from the Western Cape Province, to compare their IRMS- and SNIF-NMR-results with different vintages of Austrian wine, and to evaluate whether an unequivocal differentiation by isotope analysis is possible. As Austria is a small country, we are aware that it does not represent the entirety of Central-Europe. However, due to its heterogeneous climatic, topographic and pedogenic conditions, it might represent the isotopic characteristics of Central-European wines well beyond its borders.

## 2. Materials and Methods

Altogether, 47 South African (Republic of South Africa: RSA) wine samples (vintages 2017 and 2018) were collected and analyzed. Of these, 18 samples were small-scale vinifications [33] in triplicate by inoculated and controlled vinification of two different cultivars (Chenin blanc and Sauvignon blanc) from three different wineries around Stellenbosch, northeast of Cape Town, in 2017. The small-scale vinifications were carried out under absolutely identical laboratory conditions at Stellenbosch University, to exclude any enological influence on the results. Twenty-nine wine samples were commercial South African wine samples collected at Infruitec. These 29 samples represent large quantity wine batches of commercial wineries in South Africa.

We compare the results of our samples with published isotope data of Austrian wines, to obtain information on whether and how a differentiation between Central-European (Austrian) and South African wine is possible (see further below).

Prior to analysis, the wine samples were distilled, and thus the alcohol was separated from the wine water.

### 2.1. IRMS

The alcohol fraction is used to determine δ^13^C by injecting small quantities of each sample into an elemental analyzer connected via a ConFlo to an isotope ratio mass spectrometer (IRMS). For δ^18^O analysis, a small amount of each wine sample is filled into glass vials, which are then mounted on a heating block of a Gasbench (ThermoFisher, Bremen, Germany). There, the sample headspace of each vial is purged by a He-CO_2_ gas mixture, and the samples were left to equilibrate. After an equilibration time of a minimum of 8 h, the headspace samples were flushed by a helium stream into the IRMS (Delta V Advantage, ThermoFisher, Bremen, Germany), where the ^18^O-isotope ratio was determined. The results are reported in the conventional δ notation in ‰ versus the international standards V-PDB (Vienna-Peedee belemnite) for carbon and V-SMOW (Vienna-Standard Mean Ocean Water) for oxygen isotopes by calculating as follows:

δX‰ = ((Rsample/Rstandard) − 1) × 1000(1)

where X is ^13^C or ^18^O and R is the ratio of ^13^C/^12^C, or ^18^O/^16^O, respectively. The extended reproducibility of measurements of the carbon and oxygen isotope ratios are within ±0.3 and ±0.5‰, respectively. For quality control and comparability of results, certified standards and reference materials were also analyzed together with the wine samples (e.g., V-SMOW (δ^18^O = 0.0‰), SLAP (δ^18^O = −55.5‰) [both provided by the International Atomic Energy Agency (IAEA)]; and BCR 660 (δ^13^C = −26.72‰) and BCR 656 (δ^13^C = −26.91‰) [both produced by the Institute of Reference Materials and Measurements (IRMM)]).

### 2.2. SNIF-NMR-Analysis

Distillates of wine ethanol, obtained via an automated control distillation system (ADCS) on Cadiot distillation columns (Eurofins, Nantes, France), were controlled on water content by Karl-Fischer titration (Mettler-Toledo, Greifensee, Swiss). Before measurement the distillates were placed in Wilmad 10 mm NMR tubes (Merck, Kenilworth, NJ, USA) and supplemented by fluorine lock solution and internal certified reference standard TMU (STA-003M, IRMM, Geel, Belgium). The D/H ratios on methyl- (D/H)I and methylene- (D/H)II position of ethanol were measured using Avance Neo 600 MHz NMR spectrometer (Bruker, Karlsruhe, Germany). It was equipped with a selective double resonance deuterium probe with ^1^H decoupling at constant temperature (300 or 302 K). Each acquisition contained ten spectra with at least 50 scans to reach standard deviations and S/N (signal to noise) ratios prescribed by the method. The D/H ratios of the samples were determined using processing in EUROSPEC software (Eurofins, Nantes, France), and values were verified on the CRMs (CRM-123A, IRMM, Geel, Belgium) measured at the start and the end of each measuring session. The D/H values are expressed in ppm.

## 3. Results

**Figure 1:**
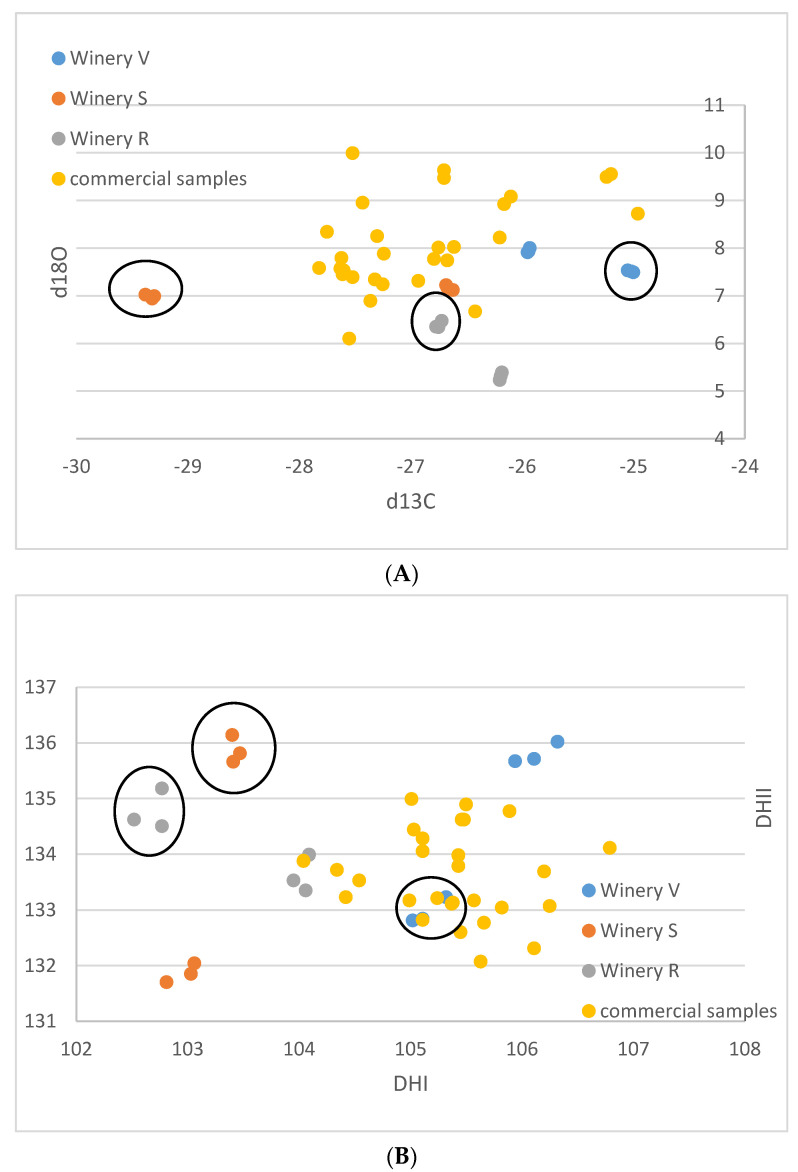

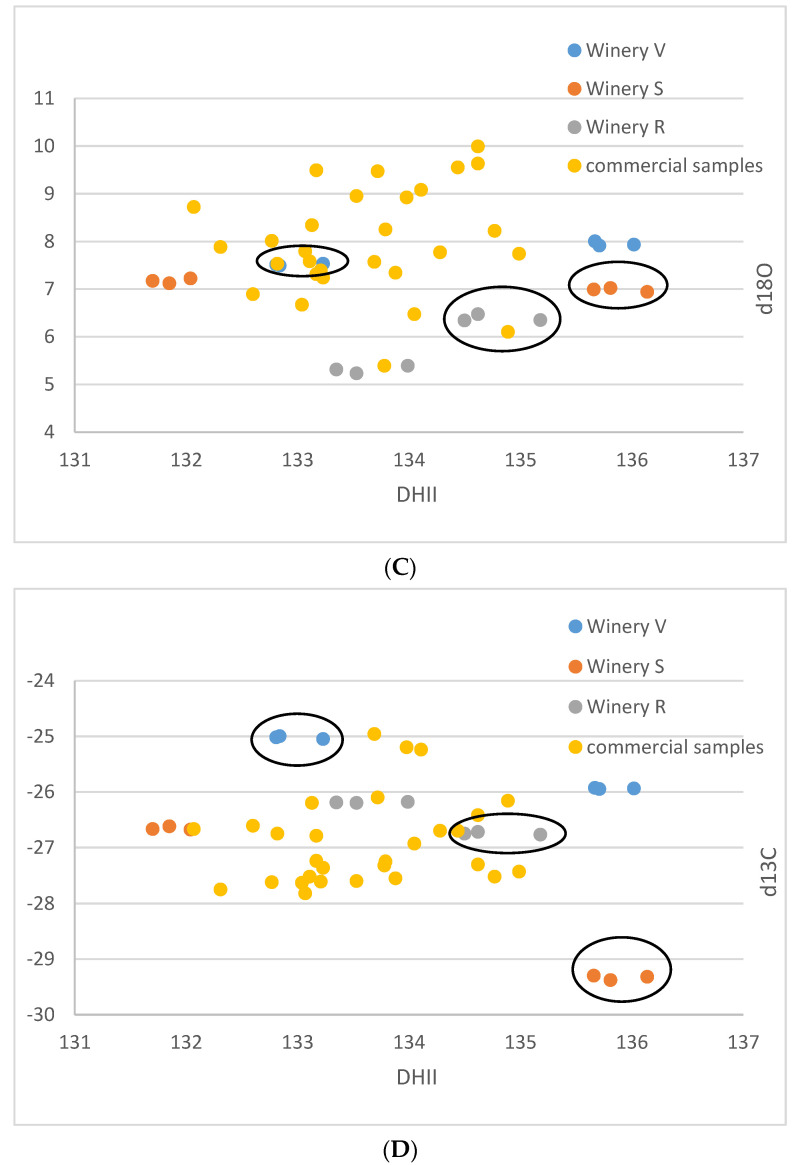

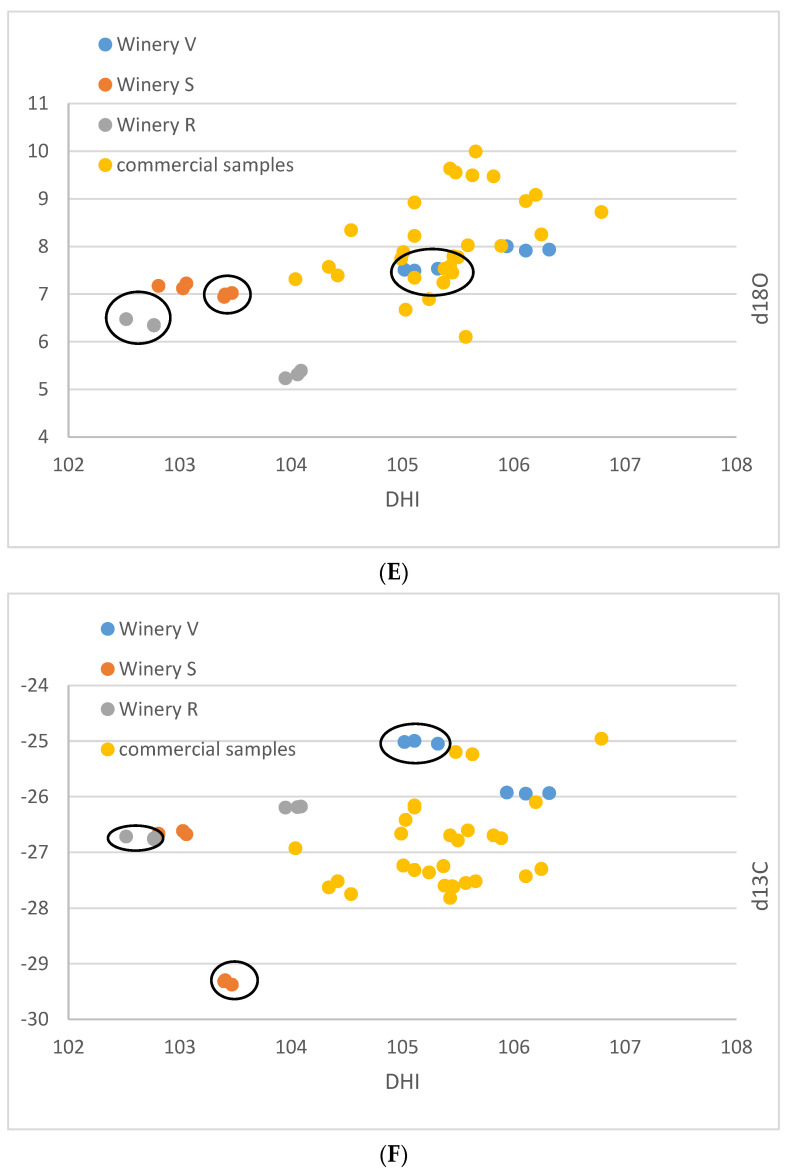
(**A**–**F**): Isotope results of the South African samples in ‰ versus V-PDB for δ^13^C, in ‰ versus V-SMOW for δ^18^O, and in ppm for (D/H)I and (D/H)II. (**A**): δ^13^C versus δ^18^O. (**B**): (D/H)I versus (D/H)II. (**C**): (D/H)II versus δ^18^O, (**D**): (D/H)II versus δ^13^C, (**E**): (D/H)I versus δ^18^O, (**F**): (D/H)I versus δ^13^C. Ellipses identify the Sauvignon Blanc from the Chenin samples of the small-scale samples of the wineries R, S, V.

For the small-scale vinifications from the Stellenbosch area, the δ^13^C-values range between −29.4 and −25.0‰, and the δ^18^O-values vary between 5.2 and 8.0‰ (Table 1). The (D/H)I values are between 102.5 and 106.3 ppm, and (D/H)II between 131.7 and 136.0 ppm (Figure 1A–F). The commercial wine samples possess δ^13^C values between −27.8 and −25.0‰, and δ^18^O values from 6.1 to 10.0‰. (D/H)I ranged from 104.0 to 106.8 ppm, and (D/H)II from 132.0 to 135.0 ppm.

**Figure 2:**
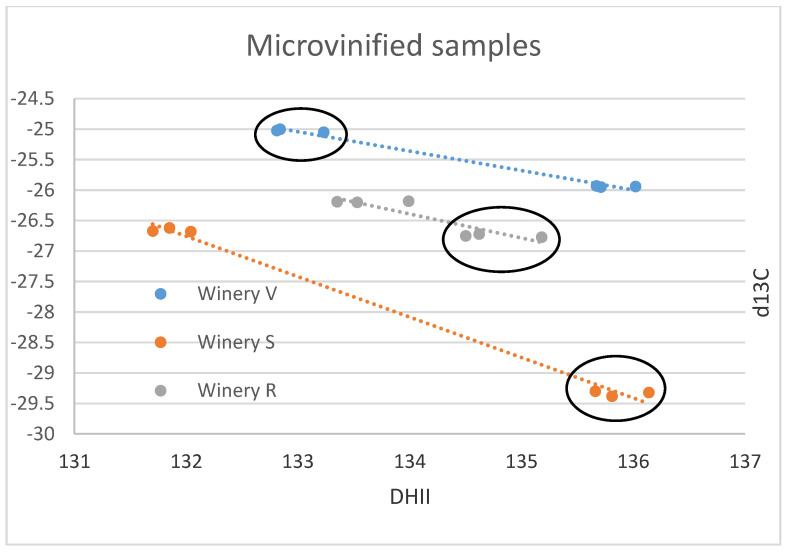
Individual wineries’ small-scale samples δ^13^C versus (D/H)II results in ‰ versus V-PDB for δ^13^C and in ppm for (D/H)II. The results of all three wineries investigated are negatively correlated. Ellipses identify the Sauvignon Blanc from the Chenin samples of the small-scale samples of the wineries R, S, V.

The results of the small-scale vinification triplicates are in almost perfect agreement (Figure 1A–F) and document exactly reproducible values under controlled vinification conditions (inoculated vinification). One triplicate (Sauvignon blanc of vinery S) possesses very low ^13^C values, notably different from the other samples. A negative correlation between δ^13^C and (D/H)II is noted (Figure 1F and Figure 2).

**Figure 3:**
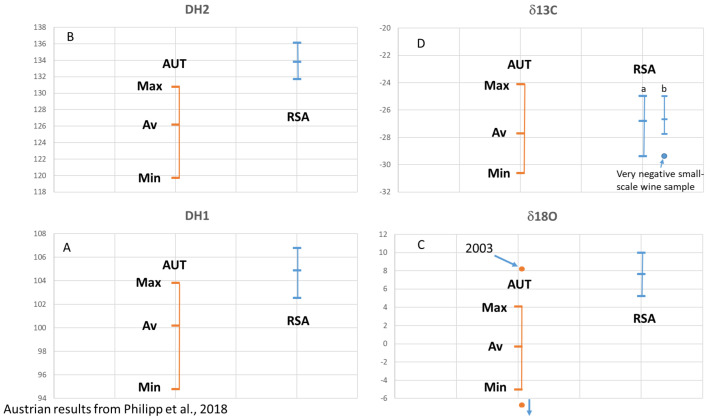
(**A**–**D**) South African (RSA) wine isotope results versus Austrian (AUT) wine isotope results for vintages 1997–2004. All values are reported in ‰ versus V-PDB for δ^13^C, in ‰ versus V-SMOW for δ^18^O, and in ppm for (D/H)I and (D/H)II. Austrian results from Philipp et al., 2018 [9]. (**C**): “2003” marks the Austrian sample from vintage 2003 with a very high δ^18^O-value. An Austrian sample with a very low δ^18^O value below −6‰ is a “dry-berry”-sample. (**D**): South African results are presented as bar (a) including all samples, and bar (b) showing separately the depleted values of small-scale samples of Sauvignon Blanc from winery S.

**Figure 4:**
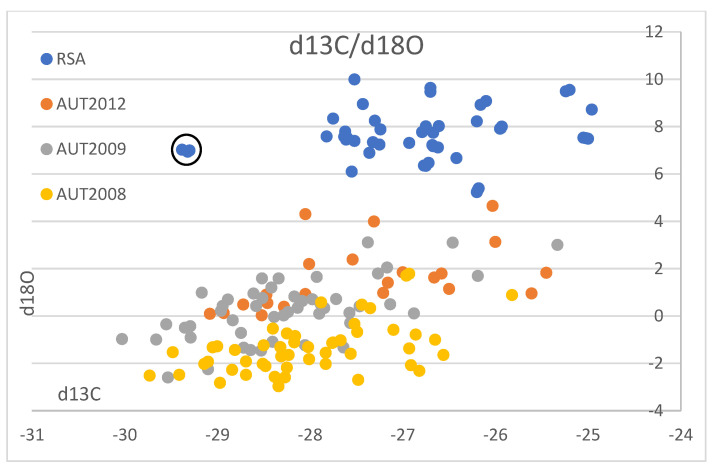
South African wine δ^13^C- and δ^18^O-isotope results and Austrian wine isotope results vintages 2008, 2009 (Horacek et al., 2021 [20]) and 2012 (Griboff et al., 2021 [31]). Values are shown in ‰ versus V-PDB for δ^13^C, and in ‰ versus V-SMOW for δ^18^O. Black circle denotes the South African small-scale vinification sample showing exceptionally low δ^13^C-values. For further explanations refer to text.

We compare our results with published data from Austrian wine samples from 1998 to 2004 (9): (D/H)I: 97.8 to 103.8 ppm, (D/H)II: 119.6 to 130.8 ppm, δ^13^C: −30.6 to −24.0‰, δ^18^O: −8.1 to 8.2‰ (Figure 3), the vintages 2008 and 2009 (20): δ^13^C: −25.3 to −30.0‰, δ^18^O: −3.0 to 3.1‰ (Figure 4), and vintage 2013 (31): δ^13^C: −29.5 to −25.5‰, δ^18^O: 0.0 to 3.1‰ (Figure 4). The results from Philipp et al., 2018 [9], and Horacek et al., 2021 [20], represent values from authentic wine samples from the EU-wine database, while the data by Griboff et al., 2021 [31] represent commercial wine samples.

## 4. Discussion

### 4.1. Irrigation in Austria (Central Europe) and Cape Province (South Africa)

For interpretation of the data, it is necessary to know about the differing irrigation practices in Austria (Central Europe) and the Western Cape Province (South Africa). In Austria and Central Europe, precipitation and soil water dominantly supply the vines, with only occasional and small-scale sprinkler irrigation that mainly uses groundwater. Moreover, a winery’s vineyards often/usually are not directly around the winery, but dispersed over some distance and are disconnected from one another.

Unlike in Austria and Central Europe, in the Western Cape intense irrigation of the vines is usually required (34) and commonly practiced, applied through dripper systems. This means that the dominant part of the water needed for the vines is supplied through irrigation. The water usually comes from water reservoirs, which are artificial lakes and ponds made by filled embankments which catch the precipitation that dominantly falls during the winter season [34]. This also means that these artificial reservoirs experience strong evaporation, as most of them are situated well within the vineyards and are unprotected against evaporation. This further means that the vineyards of individual estates, which are usually grouped together in proximity to the winery, are usually supplied by water coming from a single or several spatially closely positioned reservoirs. These reservoirs will therefore store water from the same rain events and experience similar evaporation losses, eventually resulting in irrigation water of individual wineries possessing similar isotope ratios.

Depending on where the farm is situated in the Stellenbosch area, irrigation water can also come from water schemes such as the Berg River. However, this is not the case for the wineries involved in this study (from where the small-scale wine samples come from), which all rely on dammed irrigation ponds as described above. Winery S is situated most closely to the Stellenbosch hills, profiting from the humidity coming from there and lying close to a small stream, from which small amounts of water might be pumped to the reservoirs. The other two wineries (V, R) entirely rely on rainwater reservoirs.

### 4.2. Small-Scale Vinification Wine Samples (Stellenbosch Area)

These samples are very heterogenous, as they come from six different localities of three different wineries and two different cultivars. Thus, any pattern recognized might be entirely coincidental. However, assuming that the samples from an individual winery were irrigated with similar water with respect to the isotope ratio, and experienced similar weather conditions, the following might be a valid interpretation of the observed data: The small-scale vinification samples show distinctive patterns, which enable a differentiation by the wineries. The samples from winery V are notably higher in δ^18^O than the other two wineries and show higher δ^13^C and (D/H)I(D/H) values. The samples from winery S lie in the middle, and the samples from winery R have the lowest δ^18^O-values. The δ^13^C-values of the wineries S and V are relatively similar—except that one of the triplicates of winery S shows exceptionally low δ^13^C-values below −29‰. Based on the available data, a complete differentiation of the investigated samples by wineries is possible, based on the differing δ^18^O values. However, one must keep in mind that the data are very limited, and more samples per winery might lead to overlapping values.

The small-scale samples have wider ranges of their δ^13^C- and δ^18^O-values (both exceeding 4‰) and also of the D/H-values ((D/H)I: 3.5, (D/H)II: 4.4), than the commercial samples (less than 3‰ for δ^13^C and ca. 4‰ for δ^18^O, (D/H)I: 2.8, and (D/H)II: 3), indicating that small-scale vinification might record isotopic variations that are smoothed out in large volume stored wines. Furthermore, the results of the small-scale vinification samples from the Stellenbosch area plot, on average, more on the lower side (Figure 1A,C,E) with respect to the South African wine δ^18^O values, indicating irrigation of the vineyards with water of lower isotopic ratio in the Stellenbosch area compared to other wine-producing regions of the Western Cape Region, which is in agreement with water precipitation isotope results [35]. The δ^13^C- and (D/H)II-values (except the very negative δ^13^C values of the triplicate already mentioned above) of the small-scale samples are in-line with the commercial samples, indicating similar agricultural practices concerning vineyard watering. The mentioned small-scale sample possessing very negative δ^13^C-values must have grown under complete absence of water scarcity. This is remarkable, as this value is in the range of the lowest δ^13^C-values of wine from Austria (see below), which grows in a different climatic and geographical situation. The negative correlation of δ^13^C and (D/H)II (independent from cultivar, Figure 2) seems strange at first view, as higher δ^13^C-values denote more intense drought stress, and higher (D/H)II-values indicate higher ^2^H-values of the (irrigation) water that the vines took up [8,36]. The latter implies either water from a high-temperature region, or highly evaporated water (water from a water body that has already lost a notable part of its water due to evaporation [37,38], and therefore, these parameters (drought stress and water evaporation) often correlate positively. We explain the observed negative correlation by (more intense) irrigation of the samples possessing lower δ^13^C-values with highly evaporated (surface-) water (from rainwater reservoirs) than the respective sample from the same winery with the higher δ^13^C-value. The latter samples with higher δ^13^C-ratios only (or to a greater extent) had access to (very) limited amounts of non/less fractionated soil-/groundwater and thus experienced more severe drought stress. As already noted above, the sample possessing the lowest δ^13^C-value is supposed to have been grown under a complete absence of drought stress, which only can be achieved by massive irrigation. We want to stress again, that due to the low number of samples and the assumptions made, this interpretation is preliminary and speculative. Furthermore, other explanations for the observed pattern might exist, and for a good confirmation of the interpretation, a larger data set is required.

Since the Austrian and South African wine regions are so far away from each other and are growing on different hemispheres, a comparison of identical vintages is not necessary, as the weather conditions in these two regions will follow different and independent/unrelated trends. Thus, we use literature data (Austrian reference wine samples from the years 1997 to 2004: Philipp et al., 2017 [9] (Figure 3A–D), Austrian reference wine samples from the years 2008 and 2009: Horacek et al., 2021 [20], commercial Austrian wines vintage 2012: Griboff et al., 2021 [31]) for a comparison of isotope ranges, to study, if a differentiation by isotope data is achievable (Figure 4). Generally, complete discrimination is achieved by comparison of the (D/H)II- and the δ^18^O-values of Austrian reference wine samples from the years 1997 to 2004 (Figure 3B,C), which do not overlap. The only exception was observed for one Austrian δ^18^O-result from the year 2003, which was an exceptionally hot and dry summer in Europe (also see Christoph et al., 2015 [8]). This differentiation by isotope results is confirmed by the data of Griboff et al., 2021 [31], and Horacek et al., 2021 [20], (even though the mentioned datasets do not include SNIF-NMR data) by the consistent difference in δ^18^O values (Figure 4). On the other hand, the δ^13^C-values of the Austrian and South African wines are similar and comparable, especially when also taking into account the low δ^13^C-value of the South African small-scale vinification sample (Figure 2). They indicate similar watering strategies, and thus, considering the high δ^13^C-values, also document notable drought-stress (e.g., Damiano et al., 2022 [39]) among some of the wines of both countries, which seems to be tolerated, if not (in some cases) even intended. However, and naturally because of the existing climate, drought stress is more abundant among the South African samples. In contrast, the δ^18^O values clearly show the difference in isotopes in precipitation and precipitation water between Austria (Central Europe) and South Africa (which becomes enriched in heavy isotopes due to evaporation). Consequently, the respective δ^18^O-pattern is taken up by the vines and stored in the grapes, additionally influenced by the transpiration of the vines and thus passive enrichment of the heavy oxygen and hydrogen isotopes (mainly (D/H)II) [20,29], eventually leading to the differentiation of the wines from these two countries by δ^18^O.

### 4.3. Outlook

Ongoing climate change poses a serious challenge and threat to wine production in South Africa and Austria/Central Europe. Reduced/decreasing precipitation results in smaller volumes harvested [34], unless this deficit is compensated by irrigation. Whereas in the Western Cape a challenge will become to secure the water required for irrigation, Austria/Central Europe needs to prepare to move from an agricultural practice of wine growing using precipitation towards employing irrigation. It is expected that the climatic changes and the resulting agricultural practices will lead to changes in the respective wine isotope patterns. Thus, continuing research will be necessary.

## 5. Conclusions

By analysis of stable C- and O-isotopes and (D/H)I and (D/H)II, we demonstrate the potential to differentiate South African wine samples from Austrian wines. Except for one individual Austrian wine sample of the year 2003 vintage, which was an extremely hot summer in Western and Central Europe, the South African samples show consistently higher isotope values for δ^18^O and (D/H)II (and only a small overlap for (D/H)I). We thus show that IRMS and SNIF-NMR analyses are excellent methods to differentiate between wines from South Africa and Austria (Central Europe). For δ^13^C we do not observe such strong differences between the wines from the two countries compared, even though on average the South African wines have higher values than the Austrian ones. We tentatively interpret the observed unusual negative correlation of δ^13^C and (D/H)II in small-scale wine samples from South Africa by irrigation of some of the samples with water strongly enriched in heavy isotopes due to evaporation resulting in lower δ^13^C values due to reduced/absence of drought stress and higher/increased (D/H)II-values. Also, we show that stable isotope analysis can differentiate between individual vineyards of individual wineries, when different environmental (or agricultural, in the present case most likely being water availability) conditions are present. We thus document that even wines from individual vineyards differ in their isotopic signals and therefore, can potentially be discriminated, if such claims need to be controlled. Consequently, such controls can be made from regional and national, to even individual vineyard scales. This already has been demonstrated in earlier works [4,5,40,41]. Presently, investigations have been performed on a limited number of mainly commercial samples. Future research might include more samples of exclusively authentified origin over even more vintages, as well as better discrimination within the South African wine regions. The overlap of one sample from 2003 indicates that during exceptionally hot summers in Europe (which probably will become more frequent due to global climate change), a partial overlap of wines from both investigated geographic origins might be possible. Therefore, further research and investigations will be necessary to account for potential overlapping signatures of the proxies investigated in this study in the future.

## Figures and Tables

**Table 1:** South African wine samples analyzed in this study. V, S, R: Abbreviations of individual wineries from the Stellenbosch area. SB: Sauvignon blanc, C: Chenin blanc. Colours mark individual wineries. DHI = (D/H)I, DHII = (D/H)II, d13C = δ^13^C, d18O = δ^18^O. For more explanations refer to text.

Lab No.	Winery	Cultivar	Colour	DHI	DHII	d13C	d18O
20050044	V	SB	white	105.3	133.2	−25.1	7.5
20050045	S	SB	white	103.5	135.8	−29.4	7.0
20050046	S	C	white	103.1	132.0	−26.7	7.2
20050047	V	C	white	106.3	136.0	−25.9	7.9
20050048	S	SB	white	103.4	136.1	−29.3	6.9
20050049	S	C	white	103.0	131.9	−26.6	7.1
20050050	R	SB	white	102.8	135.2	−26.8	6.4
20050051	R	SB	white	102.8	134.5	−26.8	6.3
20050052	S	C	white	102.8	131.7	−26.7	7.2
20050053	R	C	white	104.1	133.4	−26.2	5.3
20050054	S	SB	white	103.4	135.7	−29.3	7.0
20050055	R	C	white	104.0	133.5	−26.2	5.2
20050056	V	SB	white	105.0	132.8	−25.0	7.5
20050057	V	C	white	105.9	135.7	−25.9	8.0
20050058	V	SB	white	105.1	132.8	−25.0	7.5
20050059	R	C	white	104.1	134.0	−26.2	5.4
20050060	R	SB	white	102.5	134.6	−26.7	6.5
20060003			rose	105.1	133.8	−27.3	7.3
20060004			rose	104.0	134.1	−26.9	7.3
20060005			rose	105.6	133.9	−27.6	6.1
20060006			white	105.5	133.2	−26.8	7.8
20060011			red	105.1	134.9	−26.2	8.9
20060012			red	105.4	134.3	−26.7	9.6
20060013			rose	105.5	134.0	−25.2	9.6
20060014			white	105.0	134.6	−26.4	6.7
20060015			white	105.8	134.4	−26.7	9.5
20060016			white	104.3	133.0	−27.6	7.6
20060018			red	106.2	133.7	−26.1	9.1
20060019			red	106.8	133.7	−25.0	8.7
20060020			rose	105.6	134.1	−25.2	9.5
20060021			rose	105.0	132.1	−26.7	7.7
20060022			white	105.0	133.2	−27.2	7.9
20060023			red	106.1	135.0	−27.4	9.0
20060024			red	104.5	132.3	−27.8	8.3
20060025			red	105.4	133.5	−27.6	7.5
20060026			rose	105.1	133.1	−26.2	8.2
20060027			white	105.9	132.8	−26.8	8.0
20060049			red	105.7	134.8	−27.5	10.0
20060050			red	105.5	132.8	−27.6	7.8
20060051			red	106.3	134.6	−27.3	8.3
20060052			rose	105.4	133.1	−27.8	7.6
20060061			rose	105.4	133.8	−27.3	7.2
20060062			white	104.4	133.1	−27.5	7.4
20060063			white	105.2	133.2	−27.4	6.9
20060064			white	105.5	133.2	−27.6	7.5
20060065			white	105.6	132.6	−26.6	8.0
20060066	V	C	white	106.1	135.7	−26.0	7.9

V, S, R: individual wineries; C: Chenin blanc; SB: Sauvignon blanc.

## Data Availability

All new data are reported in the article.

## Supplementary Materials

The following supporting information can be downloaded at: https://www.mdpi.com/article/10.3390/foods12061175/s1, Figure S1: Map indicating Austria and South Africa.
