# Supplementary figures and images for: Differentiation of Geographic Origin of South African Wines from Austrian Wines by IRMS and SNIF-NMR
Source: Foods. 2023 Mar 10;12(6):1175. doi: 10.3390/foods12061175 (PMC10048474; doi:10.3390/foods12061175)

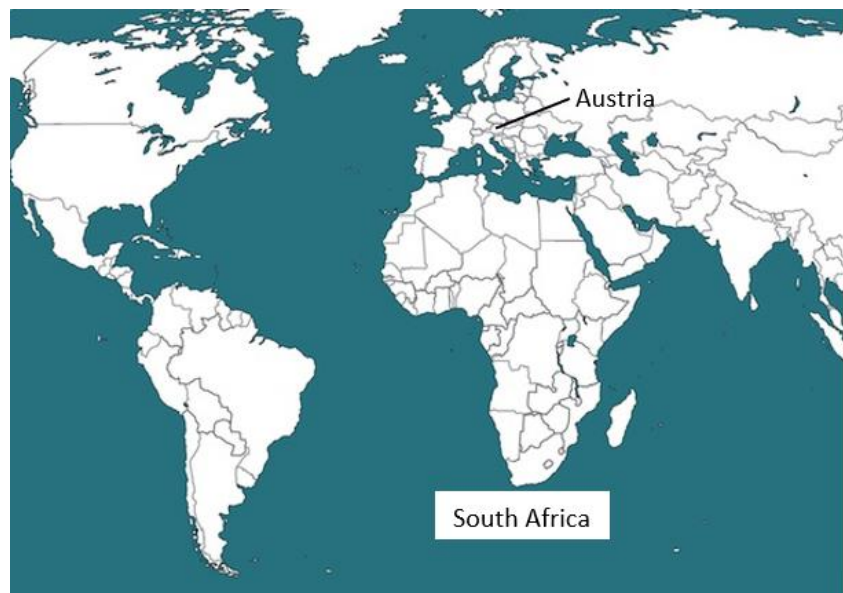

**Figure S1.** Map indicating Austria and South Africa.

Supplement: Supplementary file 1 [file foods-12-01175-s001.zip › foods-2203032-supplementary.pdf]
